# The interaction between CRY1 Polymorphism and Alternative Healthy Eating Index (AHEI) on cardiovascular risk factors in overweight women and women with obesity: a cross-sectional study

**DOI:** 10.1186/s12902-023-01429-9

**Published:** 2023-08-14

**Authors:** Fatemeh Dehghani Firouzabadi, Atieh Mirzababaei, Farideh Shiraseb, Hadith Tangestani, Khadijeh Mirzaei

**Affiliations:** 1 Department of Community Nutrition, School of Nutritional Sciences and Dietetics, Tehran University of Medical Sciences (TUMS), P.O. Box: 14155-6117, Tehran, Iran; 2 Department of Nutrition, Persian Gulf Tropical Medicine Research Center, Bushehr University of Medical Sciences, Bushehr, Iran

**Keywords:** CRY1, Alternative healthy eating index, Cardiovascular risk factors, Obesity, Overweight, Interaction

## Abstract

**Background:**

According to some studies, diet can be interaction with CRY1 polymorphism and may be related to obesity and the risk of cardiovascular diseases (CVD). So, this study examined the interaction between CRY1 polymorphism and AHEI on cardiovascular risk factors in overweight women and women with obesity.

**Methods:**

This cross-sectional study was performed on 377 Iranian women with overweight and obesity aged 18–48(BMI ≥ 25 kg/m^2^). Dietary intake was evaluated by the use of a food frequency questionnaire (FFQ) with 147 items. The AHEI was calculated based on previous studies. Anthropometric and biochemical measurements were assessed and the bioelectrical impedance analysis method was used for body analysis. The rs2287161 was genotyped by the restriction fragment length polymorphism (PCR-RFLP) method. Objects were divided into three groups based on rs2287161 genotypes.

**Results:**

Our findings determined that the prevalence of the C allele was 51.9% and the G allele was 48.0%. The mean age and BMI were 36.6 ± 9.1years and 31 ± 4 kg/m^2^ respectively. After controlling for confounders (BMI, age, total energy intake, and physical activity), this study demonstrated that there was a significant interaction between CC genotype and adherence to AHEI on odds of hyper LDL (OR = 1.94, 95% CI = 1.24–3.05, P for interaction = 0.004), hypertension (OR = 1.80, 95% CI = 1.11–2.93, P for interaction = 0.01) and hyperglycemia (OR = 1.56, 95% CI = 0.98–2.47, P for interaction = 0.05).

**Conclusions:**

This study indicated that adherence to AHEI can reduce the odds of hyper LDL, hypertension, and hyperglycemia in the CC genotype of rs2287161.

## Background

In recent years, more than 1.9 billion adults aged 18 years and older were overweight and over 650 million adults were obese [1]. In Iran, the prevalence of obesity and overweight is 27.0-38.5% and 12.6–25.9%, and the combined prevalence of them maybe 76% in some areas [2, 3]. Worryingly, women are mainly influenced by the obesity epidemic in the world [4]. There are several suggested etiologies for obesity such as over nutrition, sedentary lifestyle, variations in the gut microbiome, long sleep deprivation, special drugs inducing weight gain, and genetic factors. Also, biological and psychosocial factors, especially in women, may put them at greater risk for obesity [5, 6]. Obesity increases the risk of type 2 diabetes, cardiovascular diseases (CVDs), hypertension, dyslipidemia profile, and kinds of cancers [7, 8]. Despite both men and women are influenced by the obesity load, women showed increased CVDs risk, specifically when overweight/obese and insulin resistant [9]. Obesity risk factors are influenced by genetic factors and environmental factors.

Among environmental factors, the diet has a crucial role in obesity and CVDs development [10, 11]. In recent times, a good typical image of diet-disease associations may not be figured out from studying and analyzing single nutrients or food items and their influence. Therefore, analyzing the combination of food and nutrients may be a better contribution [12]. A novel tool, known as the alternative healthy eating index (AHEI), suggests dietary recommendations for predictive of chronic disease risk. The total score AHEI shows the overall quality of the diet and separate component scores that can be considered as a group to expose a pattern of quality regarding different dimensions of diet [13]. AHEI focuses on fat quality (e.g., omega-3 and polyunsaturated fat intake), highlights nut and legume consumption, and considers moderate alcohol consumption to be healthy regardless of disease status (e.g., diabetes). In addition, the index recommends limiting consumption of red and processed meats and added sugars (such as sugar-sweetened beverages and fruit juices). It has been reported this index is associated with reduced risks of metabolic syndrome, obesity, and CVDs risk factors such as hypertension, hyper LDL, and waist circumference [14–17].

According to recent studies, genetics is an effective factor in the etiology of chronic diseases [18]. Circadian rhythm is a biological rhythm that follows a relatively fixed period between 20 and 28 h, and its disorder tends to result in an energy imbalance [19]. Cryptochrome 1 gene (CRY1) is a regulatory gene of the circadian clock. CRY1 rs10861688 polymorphism is inversely related to the risk of abdominal obesity [20]. Besides, previous studies show the CRY1 variant is associated with CVDs risk factors such as hypertension, hypertriglyceridemia, hypercholesterolemia, hyperglycemia, and insulin resistance [21–24].

Previous studies propose the contribution of genetics, dietary, and environmental factors may play a significant role in the pathogenesis of obesity and CVDs [25, 26]. According to some recent research, diet can have an interaction with CRY1 gene polymorphism [27]. Some studies showed a high-fat diet, high-salt diet, or low-carbohydrate and high-protein diet may have an interaction with CRY1 gene variation for obesity and CVDs risk factor [24, 27–31].

Numerous studies found an inverse association between the higher score of AHEI cardiovascular risk factor and as mentioned, CRY1 rs10861688 is associated with cardiovascular disease [24, 32]. As mentioned above, previous studies found environmental factors including diet may have an interaction with CRY1 gene variation for chronic disease. By knowing demographic genotypes, we can better explain the effects of diet and its mechanisms on cardiovascular factors. Until now, no studies have investigated the interaction between CRY1 gene variation and AHEI. In this study, the interaction between CRY1 polymorphism and alternative healthy eating index (AHEI) on cardiovascular risk factors in overweight women and women with obesity was investigated.

## Method

### Study population

This cross-sectional study was carried out on 377 overweight women/ women with obesity who were referred to health centers in Tehran, Iran. Subjects were registered by the use of a simple sampling method. Inclusion criteria were age 18–48 years, being overweight or obese (body mass index (BMI) ≥ 25 kg/m^2^), not being pregnant, latescent, menopause, and no smoking and alcohol consumption. Moreover, we excluded subjects with a history of cardiovascular disease, cancer, diabetes, sustained hypertension, thyroid disease, acute or chronic infections, and liver and kidney disease. We also excluded subjects with following an unusual dietary routine, having significant weight fluctuations in the past 1 year, use of dietary supplements and weight loss medications, and participants whose conveyed daily energy intakes were 800 kcal/day or 4200 kcal/day.

### Ethical approval

Before taking part in the study, each subject completed a written informed consent form all procedures involving human subjects were approved by the Ethics Commission of Tehran University of Medical Sciences (IR.TUMS.VCR.REC.1398.051). The research was done with the support of the Tehran University of Medical Sciences (Grant number: 99.3.212.50265).

### Demographic variable

Data on age, education (illiterate, diploma, and university), marriage (single or married), occupation (employee or unemployed), economy (low, moderate, good, very good), history of weight loss in previous years (yes or no), history of family obesity (yes or no) were collected by trained nutritionist and using a demographic questionnaire.

### Physical activity

Physical activity was assessed by the use of the generally validated International Physical Activity Questionnaire (IPAQ) [33] and self-assessment. Data were shown as metabolic equivalent hours per week (MET-h/week) [34].

### Dietary assessment

Participants’ usual dietary intake for any food consumed daily, weekly, or monthly during the past year was found using a 147-item semi-quantitative food frequency questionnaire (FFQ) with a skilled nutritionist. The validity and reliability of this questionnaire have been previously reported [35]. Portion sizes of consumed foods were described in household measures and were converted to grams [36]. Nutritionist IV software, (version 7.0; N-Squared Computing, Salem, OR) modified for Iranian cuisine, was used to analyze nutrients.

### Calculation AHEI

The Alternative Healthy Eating Index (AHEI) contains 11 components including 1- fruits, 2- vegetables, 3- whole grains, 4- nuts and legumes, 5- long-chain omega-3 fatty acids, 6- polyunsaturated fatty acids, 7- sugar-sweetened beverages and fruit juice, 8- red and processed meats, 9- trans fatty acids, 10- sodium, 11- alcohol. We did not consider alcohol intake due to a lack of information. The consumption of fruits, vegetables, whole grains, nuts and legumes, long-chain omega-3 fatty acids, and polyunsaturated fatty acids were scored 10 and 0 in the highest and lowest intake, respectively. Also, the consumption of sugar-sweetened beverages and fruit juice, red and processed meats, trans fatty acids, and sodium were scored 10 and 0 in the lowest and highest consumption. Therefore, the AHEI score ranges from 0 (non-adherence) to 100 (perfect adherence) [13]. Participants were categorized into four groups based on adherence to AHEI.

### Anthropometric measurements

Weight was measured by using a digital scale (BC 08, Beurer, Germany) with a sensitivity of 0.1 kg. Height was measured by the use of a non-flexible tape measure and recorded to the nearest 0.1 cm. The measurement was performed while the participants were in a standing position, with light clothes and no shoes. Waist circumference (WC) and hip circumference (HC) were measured in the central point of the iliac crest and rib cage with flexible tape with an accuracy of 0.1 cm. The waist-to-hip ratio was calculated by dividing WC by HC. BMI was also calculated by use of the equation “weight (kg) / height^2^ (m^2^).

### Complete body composition analysis

Body composition of subjects was evaluated by Body Composition Analyzer BC-418MA- In Body (United Kingdom). This Bioelectrical Impedance Analyzer (BIA) is considered to send a very weak electric current to assess the body impedance (electrical resistance). We followed all of the following instructions for an accurate measurement. To avoid possible discrepancies in the measured values, participants were asked not to exercise vigorously, do not use any electrical devices, and not consume too much fluid or food before evaluating their body composition. Body composition analysis was performed while participants were fasting and urinating [37].

 Blood pressure.

Blood pressure was measured after the participants had been at rest for 10 min. Hypertension was defined as systolic blood pressure ≥ 130 mm Hg and/or diastolic blood pressure ≥ 85 mm Hg [38].

### Biochemical assessment

To assessment fasting serum glucose, insulin, and serum lipids, enzymes blood samples were assessed after 8–12 h of fasting at the Nutrition and Biochemistry Laboratory of the school of Nutritional and Dietetics at Tehran University of medical sciences. Fasting blood sugar (FBS) was measured on the day of blood accumulation by phenol-4-aminoanthyrine peroxidase (GOD / PAP) glucose oxidase. The concentration of serum triglycerides (TG) by use of the kits triacylglycerol (test Pars Inc, Tehran, Iran) glycerol-3-phosphate oxidase method using phenol 4-Mynvanty Pirin peroxidase (GPOPAP) were assessed. Total cholesterol (total-chol) levels were measured by the cholesterol oxidase Phenol 4-Aminoantipyrine Peroxidase (CHOD-PAP), and low-density lipoprotein (LDL) and high-density lipoprotein (HDL) was assessed by the direct method and immunoinhibition. Serum insulin concentrations were analyzed through the enzyme-linked immunosorbent assay (ELISA) method (Human insulin ELISA kit, DRG Pharmaceuticals, GmbH, Germany) [37]. Hypertriglyceridemia were defined as fasting serum TG ≥ 1.69 mmol/L. Hypo HDL was defined as HDL < 1.29 mmol/L. Hypercholesterolemia was defined as total cholesterol > 5.18 mmol/L Hyper LDL was defined as LDL cholesterol > 2.59 mmol/L. Abnormal fasting blood glucose concentration was defined as ≥ 5.55 mmol/L.

### The HOMA-IR calculation

Homeostatic model assessment-insulin resistance (HOMA-IR), was determined based on the following equation: [fasting plasma glucose (mmol/l) × fasting plasma insulin (mIU/l)]/22.5 [39].

### DNA extraction

All participants from whom deoxyribonucleic acid (DNA) samples were accessible, were evaluated to be genotyped for the rs2287161. Genomic DNA extraction from whole blood samples was performed using Mini Columns (Type G Exgene; Genall; Korea) based on the manufacturer’s protocol. The concentration and quality of the extracted DNA were assessed by the use of a NanoDrop ND-2000 spectrometer. The rs2287161 (major allele: C; minor allele: G) was genotyped by polymerase chain reaction-restricted length polymorphism (PCR–RFLP) technique. PCR applied the following primers: forward 5′-GGAACAGTGATTGGCTCTATCT − 3′; reverse 5′-GGTCCTCGGTCTCAAGAAG-3′. PCR reactions were done in a final volume of 20 µl include of 2 µl primers, 1 µl extracted DNA,7 µl distilled water, and 10 µl Taq DNA Polymerase Master Mix (Amplicon; Denmark) with the next conditions in a DNA thermocycler: The DNA templates were denatured at 94° C for 4 min; amplification contained 35 cycles at 94 °C, 58 and 72 °C (each stage for 30 s), with a final extension at 72 °C for 7 min. Amplified DNA (10 µl) was mixed with 2 µl of the DRI restriction enzyme (Thermo Fisher Scientific; USA) at 37 ° C. To ensure the PCR process and amplification of the desired parts, PCR product electrophoresis was performed on the agarose gel. Fragments including three possible genotypes were then determined: uncut homozygous GG (107 bp), cut heterozygous GC (107, 48, and 226 bp), and cut homozygous CC (155 and 226 bp) [40].

### Statistical analysis

Normality distribution was analyzed by applying Kolmogorov–Smirnov’s test. Information of continue characteristics was shown as the mean ± SD and information of categorical characteristics was reported as a number and percentage. A comparison of continuous and categorical variables across the quartiles of AHEI or genotypes was done by the use of one-way analysis of variance (ANOVA) and chi-square, respectively. Also, we adjusted variables for confounders (age, energy intake, BMI, and physical activity) by Analysis Of Covariance (ANCOVA). Genotypes were recorded based on risk allele: code 0 for GG, 1 for GC, and 2 for CC genotype. In order to examine the interactions between rs2287161 genotype and quartiles of AHEI on odds of CVDs risk factors, the participants were grouped based on CRY1 genotypes: group 1 with CC genotype (*n* = 122), group 2 with GC genotype (*n* = 148), and group 3 with GG genotype (*n* = 107). The binary logistic regression model was used to analyze potential interactions between rs2287161 genotype and quartiles of AHEI on odds of CVDs risk factors before and after adjustment for confounders (BMI, age, total energy intake physical activity, and socioeconomic status). Also, the remaining method was used to control energy in the AHEI. Moreover, we calculated the percentage of cardiovascular risk factors across CC, GC, and GG genotypes by chi-square test. Data were analyzed using IBM SPSS version 23.0 (SPSS, Chicago, IL, USA). *P* < 0.05 was considered statistically significant.

## Result

### Study population characteristics

377 overweight women or women with obesity in this cross-sectional research were studied. The means and standard deviation (SD) of age, weight, and BMI of participants were 36.67 ± 9.10 years, 81.29 ± 12.43 kg, and 31.26 ± 4.29 kg/m^2^, respectively (Table 1). The frequencies of G and C alleles of rs2287161 were 48.0% and 51.9%, respectively. The total prevalence of rs2287161 genotypes was 26.5%, 36.6%, and 30.2% for GG, GC, and CC respectively (Table 2).

**Table 1:** Study population characteristics

	Min	Max	Men or frequency	SD
**Demography**				
Age (year)	18.00	48.00	36.67	9.10
Weight (kg)	59.50	136.60	81.29	12.43
Height (cm)	142.00	179.00	161	5.87
Physical activity(MET)	40.00	19194.00	1202.05	2085.34
**Body composition**				
BMI(kg/m^2^)	24.20	49.60	31.26	4.29
SMM(kg)	17.30	37.90	25.56	3.44
Fat percentage %	15.00	56.20	42.22	5.46
FFM (kg)	33.40	67.70	46.52	5.71
BFM(kg)	19.40	74.20	34.74	8.75
WC(cm)	79.60	136.00	99.61	10.07
WHR	0.81	92.00	1.16	4.74
FFMI	13.60	147.80	18.19	6.67
FMI	6.90	26.90	13.44	3.39
**Blood pressure**				
SBP(mmHg)	13.00	159.00	111.38	14.80
DBP(mmHg)	8.00	111.00	77.60	10.40
Pulse	49.00	125.00	79.76	10.59
**Blood parameters**				
TG(mg/dl)	37.00	328.00	118.10	58.88
HDL(mg/dl)	18.00	87.00	46.58	10.86
LDL(mg/dl)	34.00	156.00	95.30	24.12
Cholesterol(mg/dl)	104.00	344.00	185.30	35.77
AST(mg/dl)	6.00	60.00	18.05	7.75
ALT(mg/dl)	4.00	98.00	19.46	13.83
FBS(mg/dl)	67.00	137.00	87.49	9.64
Insulin (mIU/ml)	0.60	1.99	1.21	0.23
HOMA-IR	1.29	9.19	3.35	1.27
**Marital status**				
Single	109(27.0%)			
Married	286(70.8%)			
**Education**				
Illiterate	4(1.0%)			
Diploma	49(12.1%)			
University	342(84.7%)			
**Level of economy**				
Low	40(9.9%)			
Moderate	167(41.3%)			
Good	155(38.4%)			
Very good	20(5.0%)			
**Occupation**				
Unemployed	|250(61.9%)			
Employed	142(35.1%)			
**History of weight loss**				
Yes	196(48.5%)			
No	168(41.6%)			
**History of family obesity**				
Yes	267(66.1%)			
No	109(26.7%)			

Variables is presented by mean ± SD for continuous variables and frequency for categorical variables

*Abbreviations*: *SD *Standard deviation, *BMI *Body mass index, *TG *Triglyceride, *LDL *Low density lipoprotein, *HDL *High density lipoprotein, *AST *Aspartate aminotransferase, *ALT *Alanine aminotransferase, *FBS *Fasting blood sugar, *SBP *Systolic blood pressure, *DBP *Diastolic Blood Pressure, *BFM *Body fat mass, *FFM *Fat free mass, *SMM *Skeletal muscle mass, *WC *Waist circumference, *WHR *Waist height ratio, *FMI *Fat mass index, *FFMI *Fat free mass index

**Table 2:** rs2287161 genotypes and allelic variants of study population

	**Genotypes frequency**			**Alleles frequency**	
**rs2287161 genotypes**	**CC**	**GC**	**GG**	**C**	**G**
	(*n* = 122) 32.4%	(*n* = 148)39.3%	(*n* = 107)28.4%	51.9(*n* = 196)%	48.0(*n* = 181)

### Association between characteristics of the study population and rs2287161 genotypes

The whole number of 377 Iranian women were grouped according to rs2287161 genotypes and placed into three groups: GG genotype (*n* = 107), GC genotype (*n* = 148), and CC genotype (*n* = 122) (Table 3). After grouping the genotypes, we indicated a significant differences of genotypes in body mass index (BMI) (*P* = 0.04), fat mass index (FMI) (*P* = 0.05), high-density lipoprotein (HDL) (*P* = 0.03), alanine aminotransferase (ALT) (*P* = 0.05), pulse (*P* = 0.03), economic status (*P* = 0.007). Also, we obtained significant differences between genotypes for age (*P* = 0.04), physical activity (*P* = 0.04), fasting blood sugar (FBS) (*P* = 0.05), and education (*P* = 0.01) after adjustment for confounders (BMI, age, total energy intake, and physical activity).

**Table 3:** Characteristics of study population according to rs2287161 genotypes

rs2287161 genotypes	GG (*n* = 107) Mean ± SD	CG (*n* = 148) Mean ± SD	CC (*n* = 122) Mean ± SD	*P*-value	*P*-value*
**Demography**					
Age (year)	35.03 ± 8.30	36.92 ± 9.49	37.77 ± 9.30	0.07	0.04**
Weight(kg)	80.01 ± 11.57	79.86 ± 12.38	83.00 ± 12.06	0.07	0.10^a^
Height(cm)	161.81 ± 5.66	160.70 ± 5.42	161.09 ± 6.09	0.31	0.35
Physical activity(METs)	911.52 + 827.49	1227.85 + 2244.90	1569.51 + 2867.89	0.19	0.04**
**Body composition**					
BMI(kg/m^2^)	30.53 ± 4.04	31.00 ± 4.06	31.89 ± 4.42	**0.04**	0.28^**^
Fat percentage %	41.43 ± 4.86	42.54 + 5.27	42.40 ± 5.81	0.22	0.55^a^
BFM(kg)	33.48 ± 7.88	34.54 ± 8.89	35.59 ± 8.51	0.17	0.57^a^
FFM(kg)	46.46 ± 5.58	45.77 ± 5.65	47.07 ± 5.58	0.17	0.35^a^
SMM(kg)	25.45 ± 3.32	25.09 ± 3.37	25.96 ± 3.44	0.11	0.35^a^
WHR	0.93 ± 0.05	0.93 ± 0.05	0.94 ± 0.04	0.48	0.16^a^
WC(cm)	98.30 ± 9.39	99.03 ± 10.38	100.78 ± 9.46	0.14	0.10^a^
FFMI	17.71 ± 1.62	18.58 ± 10.90	18.11 ± 1.54	0.60	0.97^a^
FMI	12.80 ± 3.03	13.40 ± 3.29	13.84 ± 3.50	**0.05**	0.74^a^
**Blood parameters**					
Cholesterol(mg/dl)	187.22 ± 38.26	185.17 ± 34.81	182.72 ± 34.20	0.74	0.16
TG(mg/dl)	117.81 ± 70.57	124.86 ± 72.14	123.89 ± 70.42	0.79	0.69
HDL(mg/dl)	46.16 ± 9.92	48.58 ± 12.06	44.13 ± 10.54	**0.03**	0.60
LDL(mg/dl)	95.14 ± 23.94	95.92 ± 24.24	93.44 ± 24.67	0.80	0.93
AST(mg/dl)	18.90 ± 8.68	16.52 ± 4.99	18.95 ± 9.81	0.07	0.81
ALT(mg/dl)	21.64 ± 16.50	16.67 ± 8.05	20.77 ± 16.95	**0.05**	0.59
FBS(mg/dl)	85.71 ± 7.97	87.63 ± 9.03	89.31 ± 11.65	0.07	**0.05**
Insulin (mIU/ml)	1.21 ± 0.22	1.20 ± 0.24	1.25 ± 0.23	0.37	0.46
HOMA-IR	1.18 ± 0.14	1.38 ± 0.14	3.36 ± 1.26	0.51	0.87
**Blood pressure**					
SBP (mmHg)	109.23 ± 16.62	112.58 ± 13.55	111.21 ± 15.20	0.32	0.94
DBP (mmHg)	77.33 ± 12.31	77.41 ± 10.17	77.06 ± 9.34	0.97	0.27
Pulse	82.19 ± 11.45	77.91 ± 9.31	79.14 ± 11.59	**0.03**	0.50
**Marital status**					
Single	34.6%	36.5%	28.8%	0.22	0.20
Married	25.7%	40.4%	34.0%		
**Education**					
Illiterate	50.0%	50.0%	0.0%	0.28	**0.01**
Diploma	38.3%	31.9%	29.8%		
University	26.4%	40.3%	33.3%		
**Economy**					
Low	15.8%	42.1%	42.1%	**0**.**007**	0.35
Moderate	35.5%	41.9%	22.6%		
Good	22.2%	36.1%	41.7%		
Very good	36.8%	31.6%	36.1%		
**Occupation**					
Unemployed	30.5%	39.1%	30.5%	0.35	0.25
Employed	24.1%	39.8%	36.1%		
**History of weight loos**					
Yes	31.8%	35.2%	33.0%	0.19	0.35
No	24.5%	44.0%	31.4%		
**History of family obesity**					
Yes	28.9%	40.7%	30.5%	0.86	0.72
No	29.1%	37.9%	33.0%		

Variables is presented by mean ± SD for continuous variables and frequency for categorical variables

*Abbreviations*: *SD *Standard deviation, *BMI *Body mass index, *TG *Triglyceride, *LDL *Low density lipoprotein, *HDL *High density lipoprotein, *AST *Aspartate aminotransferase, *ALT *Alanine aminotransferase, *FBS *Fasting blood sugar, *SBP *Systolic blood pressure, *DBP *Diastolic Blood Pressure, *BFM *Body fat mass, *FFM *Fat free mass, *SMM *Skeletal muscle mass, *WC *Waist circumference, *WHR *Waist height ratio, *FMI *Fat mass index, *FFMI *Fat free mass index

*P* values resulted from the analysis of one-way ANOVA for continuous variables and chi-square test for categorical variables. We also performed a Tukey test to compare each genotype with other types for continuous variables

*P-value is found by ANCOVA and adjusted for age, BMI, physical activity, and total energy intake

** Put out the collinear variable from the GLM as confounders

^a^BMI considered as collinear and this variable adjusted for age, physical activity, and total energy intake

### Dietary intake across rs2287161 genotypes

We found a marginally significant difference in vegetables (*P* = 0.06) across rs2287161 genotypes. Also, after controlling for Energy intake across rs2287161 genotypes, there were significant differences in vegetables. No significant differences were observed for other variables (Table 4).

**Table 4:** Dietary intake of study population according to rs2287161 genotypes

rs2287161 genotypes	GG (*n* = 107) Mean ± SD	CG (*n* = 148) Mean ± SD	CC (*n* = 122) Mean ± SD	*P*-value	*P*-value*
**AHEI**	51.18 + 9.73	51.88 + 8.46	52.72 + 9.58	0.56	0.28
**Component of AHEI**					
Fruits (g/d)	529.69 + 313.57	548.06 + 350.70	538.22 + 358.02	0.93	0.63
Vegetables (g/d)	421.27 + 261.59	413.65 + 219.38	497.36 + 291.82	0.06	0.03
Whole grains (g/d)	7.61 + 10.09	7.08 + 9.61	8.27 + 11.95	0.75	0.64
red meat (g/d)	27.31 + 25.30	28.16 + 26.31	30.07 + 27.10	0.72	0.28
Sweet beverage (g/d)	76.80 + 198.96	50.41 + 83.71	57.39 + 103.12	0.29	0.34
Omega3 (g/d)	0.14 + 0.17	0.11 + 0.14	0.13 + 0.15	0.42	0.87
Poly Unsaturated(g/d)	20.70 + 9.09	19.38 + 8.31	20.59 + 9.34	0.41	0.74
Trans fatty acid (g/d)	0.0008 + 0.001	0.0005 + 0.001	0.0009 + 0.002	0.13	0.61
Sodium (g/d)	4583.29 + 1607.19	4350.81 + 1558.00	4788.48 + 2100.73	0.13	0.10
**Macronutrient**					
Energy (kcal)	2739.85 + 827.69	2595.70 + 798.87	2683.54 + 798.03	0.37	0.65
Carbohydrate (g/d)	392.12 + 130.94	356.09 + 123.19	380.54 + 119.98	0.23	0.59
Protein (g/d)	93.83 + 32.08	91.01 + 31.99	93.15 + 31.11	0.76	0.65
Fat (g/d)	97.63 + 33.70	94.10 + 32.51	96.53 + 36.46	0.70	0.73
**Fiber**					
Fiber (g/d)	50.18 + 21.64	47.58 + 21.81	46.97 + 20.97	0.50	0.29
**Vitamins**					
B1(mg/d)	2.18 + 0.78	2.16 + 0.77	2.14 + 0.64	0.92	0.97
B2 (mg/d)	2.35 + 0.90	2.33 + 0.93	2.24 + 0.76	0.60	0.37
B3 (mg/d)	27.33 + 10.73	26.14 + 9.98	26.87 + 10.04	0.65	0.92
B6 (mg/d)	2.26 + 0.79	2.17 + 0.75	2.25 + 0.73	0.62	0.62
B9 (µg/d)	642.70 + 196.04	612.07 + 197.89	635.73 + 182.12	0.41	0.77
B12 (µg/d)	4.52 + 2.75	4.55 + 2.82	4.15 + 1.86	0.39	0.30
C (mg/g)	202.17 + 117.88	186.85 + 133.44	185.62 + 95.84	0.51	0.36
D (µg/d)	2.02 + 1.54	1.99 + 1.72	1.99 + 1.48	0.98	0.98
E (mg/d)	17.59 + 9.10	16.82 + 8.85	17.22 + 8.78	0.79	0.92
A (RAE/day)	785.96 + 420.29	762.56 + 399.75	778.83 + 421.91	0.90	0.90
**Minerals**					
Calcium (mg)	1304.11 + 565.12	1295.81 + 547.30	1270.23 + 504.71	0.88	0.80
Iron (mg)	27.69 + 24.00	26.46 + 20.53	27.72 + 20.13	0.86	0.80
Magnesium (mg)	485.46 + 180.27	470.47 + 174.99	495.76 + 162.01	0.48	0.14
Zinc (mg)	13.60 + 5.02	13.42 + 4.90	13.86 + 4.81	0.76	0.14
Copper (mg)	2.11 + 0.87	1.99 + 0.70	2.07 + 0.70	0.40	0.91
Potassium (mg)	4710.51 + 1808.70	4454.22 + 1720.92	4638.04 + 1663.34	0.48	0.90

Variables is presented by mean ± SD

*Abbreviation*: *AHEI *Alternative healthy eating index; Retinol activity equivalents

*P* values resulted from the analysis of one-way ANOVA

*P*-value* is obtained by ANCOVA after adjustment for calories intake

### Association between characteristics of the study population and AHEI

 All subjects were assessed across quartiles of AHEI (Table 5). We found a significant difference for weight (*P* = 0.01), BMI (*P* = 0.01), fat free mass (FFM) (*P* = 0.03), skeletal muscle mass (SMM) (*P* = 0.03), waist circumference (WC) (*P* = 0.02), low-density lipoprotein (LDL) (*P* = 0.05), systolic blood pressure (SBP) (*P* = 0.001) and diastolic blood pressure (DBP) (*P* = 0.02) across quartiles of AHEI. Also, we observed a significant difference for age (*P* = 0.05) and physical activity (*P* = 0.04) after adjustment for confounding variables (BMI, age, total energy intake, and physical activity).

**Table 5:** Evaluation continuous variables and categorical variables across the quartiles of the AHEI

AHEI	Q1 (*N* = 72) Mean ± SD	Q2 (*N* = 73) Mean ± SD	Q3 (*N* = 73) Mean ± SD	Q4 (*N* = 73) Mean ± SD	*P*-value	*P*-value*
**Demography**						
Age (year)	34.62 ± 8.92	36.93 ± 8.58	36.87 ± 8.89	37.63 ± 7.45	0.16	0.05**
Weight(kg)	79.09 ± 12.35	79.81 ± 11.95	84.70 ± 13.60	79.19 ± 10.04	**0.01**	0.63^a^
Height(cm)	162.23 ± 6.45	160.73 ± 5.47	161.43 ± 5.99	160.75 ± 5.75	0.37	0.50
Physical activity(MET)	770.57 ± 806.18	1142.89 ± 1728.47	1394.50 ± 2591.67	1470.54 ± 2649.21	0.24	0.04**
**Body composition**						
BMI(kg/m^2^)	30.19 ± 4.32	30.87 ± 4.36	32.38 ± 4.74	30.73 ± 3.57	**0.01**	0.47^**^
Fat percentage %	41.04 ± 5.19	41.74 ± 5.50	41.74 ± 6.48	41.53 ± 4.91	0.86	0.26^a^
BFM(kg)	33.06 ± 8.56	33.58 ± 8.77	36.11 ± 9.88	33.25 ± 7.06	0.11	0.06^a^
FFM(kg)	46.52 ± 5.71	45.94 ± 5.21	48.39 ± 5.77	46.25 ± 5.37	**0.03**	0.66^a^
SMM(kg)	25.49 ± 3.35	25.15 ± 3.06	26.63 ± 3.39	25.44 ± 3.25	**0.03**	0.50^a^
WHR	2.21 ± 10.80	0.92 ± 0.05	0.94 ± 0.05	0.92 ± 0.05	0.38	0.37^a^
WC(cm)	97.75 ± 9.55	98.04 ± 10.15	102.09 ± 10.94	97.93 ± 8.93	**0.02**	0.51^a^
FFMI	17.59 ± 1.47	17.80 ± 1.44	20.35 ± 15.30	17.86 ± 1.44	0.10	0.56^a^
FMI	12.59 ± 3.27	13.06 ± 3.44	13.93 ± 3.78	13.03 ± 2.96	0.12	0.48^a^
**Blood parameters**						
Cholesterol(mg/dl)	179.63 ± 40.73	184.74 ± 33.75	191.04 ± 38.04	184.84 ± 32.54	0.40	0.84
TG(mg/dl)	106.57 ± 54.49	123.50 ± 69.76	135.63 ± 72.22	123.04 ± 79.78	0.16	0.21
HDL(mg/dl)	46.00 ± 10.68	47.04 ± 10.62	47.34 ± 12.25	46.78 ± 9.97	0.92	0.59
LDL(mg/dl)	89.03 ± 26.83	95.56 ± 22.42	100.16 ± 23.84	94.96 ± 23.07	0.09	0.39
AST(mg/dl)	16.78 ± 6.35	17.53 ± 5.71	20.01 ± 10.03	16.92 ± 6.27	**0.05**	0.25
ALT(mg/dl)	17.68 ± 11.38	18.01 ± 9.20	22.86 ± 17.79	18.06 ± 11.60	0.08	0.37
FBS(mg/dl)	87.80 ± 8.64	86.88 ± 12.19	87.85 ± 9.74	87.46 ± 7.56	0.94	0.35
Insulin(mIU/dl)	1.22 ± 0.21	1.25 ± 0.26	1.24 + 0.25	1.22 ± 0.10	0.85	0.73
HOMAIR index	3.06 ± 1.07	3.26 ± 1.53	3.45 + 1.52	4.03 ± 1.24	0.44	0.54
**Blood pressure**						
SBP (mmHg)	108.97 ± 13.43	109.87 ± 12.15	117.36 ± 13.20	110.63 ± 14.83	**0.001**	0.38
DBP (mmHg)	75.95 ± 9.39	78.36 ± 8.19	80.44 ± 9.15	76.39 ± 11.10	**0.02**	0.80
Pulse	80.49 ± 9.24	80.88 ± 9.63	80.19 + 12.80	77.51 ± 10.36	0.26	0.14
**Marital status**						
Single	34.4%	21.9%	21.9%	21.9%	0.23	0.33
Married	21.8%	26.2%	26.2%	25.8%		
**Education**						
Illiterate	33.3%	0.0%	66.7%	0.0%	0.21	0.92
Diploma	12.5%	25.00%	35.00%	27.5%		
University	26.4%	25.6%	23.2%	24.8%		
**Economy**						
Low	6.7%	26.7%	26.7%	40.0%	0.18	0.16
Moderate	25.4%	30.0%	23.8%	20.8%		
Good	27.8%	20.4%	26.9%	25.0%		
Very good	44.4%	22.2%	22.2%	11.1%		
**Occupation**						
Unemployed	24.6%	24.1%	27.2%	24.1%	0.77	0.95
Employed	24.5%	28.7%	22.3%	24.5%		
**History of weight loos**						
Yes	25.2%	25.2%	27.2%	22.5%	0.79	0.70
No	25.6%	25.6%	22.6%	26.3%		
**History of family obesity**						
Yes	26.1%	24.8%	24.3%	24.8%	0.86	0.55
No	22.2%	27.8%	27.8%	22.2%		

Variables is presented by mean+SD for continuous variables and frequency for categorical variables

*Abbreviations*: *SD *Standard deviation, *BMI *Body mass index, *TG *Triglyceride, *LDL *Low density lipoprotein, *HDL *High density lipoprotein, *AST *Aspartate aminotransferase, *ALT *Alanine aminotransferase, *FBS *Fasting blood sugar, *SBP *Systolic blood pressure, *DBP *Diastolic Blood Pressure, *BFM *Body fat mass, *FFM *Fat free mass, *SMM *Skeletal muscle mass, *WC *Waist circumference, *WHR *Waist height ratio, *FMI *Fat mass index, *FFMI *Fat free mass index

*P* values resulted from the analysis of one-way ANOVA for continuous variables and chi-square test for categorical variables. We also performed a Tukey test to compare each genotype with other types for continuous variables

*P-valueis found by ANCOVA and adjusted for age, BMI, physical activity, and total energy intake.7

** Put out the collinear variable from the GLM as confounders

^a^BMI considered as collinear and this variable adjusted for Age, physical activity, and total energy intake

### Interaction between AHEI and CRY1 gene variants on cardiovascular risk factors

**Fig. 1:**
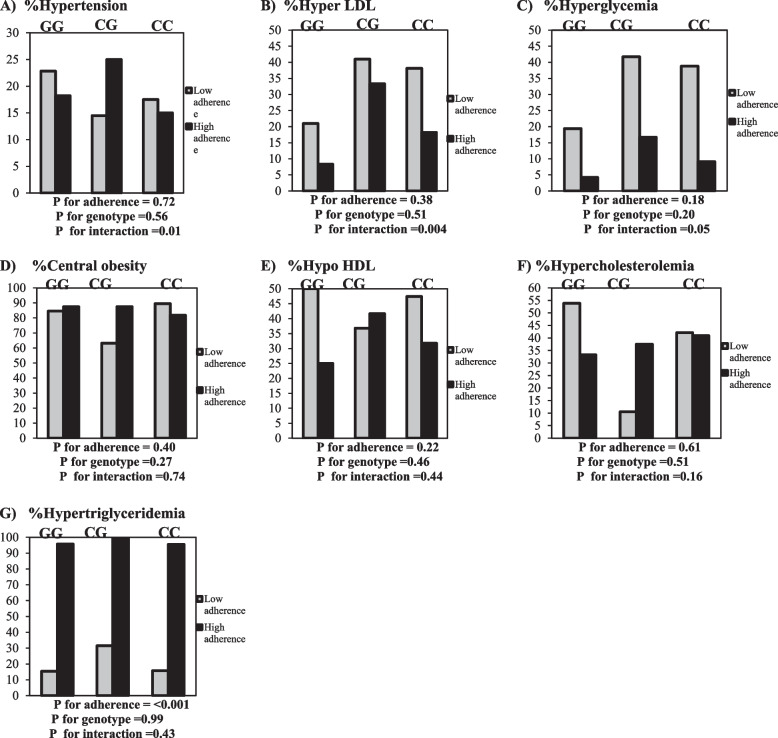
Percentage of cardiovascular risk factors across CC, GC, and GG genotypes base on low and high adherence to AHEI. **A**. Percentage of hypertension in low adherence across CC, GC, and GG genotypes were respiratory 17.5%, 14.5%, and 22.8%. Percentage of hyperglycemia in high adherence across CC, GC, and GG genotypes were respiratory 15.0%, 25.0%, and 18.2%. **B**. Percentage of hyper LDL in low adherence across CC, GC, and GG genotypes were respiratory 38.1%, 41.0%, and 21.0%. Percentage of hyper LDL in high adherence across CC, GC, and GG genotypes were respiratory 18.2%, 33.3%, and 8.3%.**C**. Percentage of hyperglycemia in low adherence across CC, GC, and GG genotypes were respiratory 38.8%, 41.7%, and 19.4%. Percentage of hyperglycemia in high adherence across CC, GC, and GG genotypes were respiratory 9.1%, 16.7%, and 4.2%. **D**. The percentage of central obesity in low adherence across CC, GC, and GG genotypes were respiratory 89.5%, 63.2%, and 84.6%. Percentage of central obesity in high adherence across CC, GC, and GG genotypes were respiratory 81.8%, 87.5%, and 87.5%. **E**. Percentage of hypo HDLin low adherence across CC, GC, and GG genotypes were respiratory 47.4%, 36.8%, and 50.0%. Percentage of hypo HDL in high adherence across CC, GC, and GG genotypes were respiratory 31.8%, 41.7%, and 25.0%. **F**. Percentage of hypercholesterolemia in low adherence across CC, GC, and GG genotypes were respiratory 42.1%, 10.5%, and 53.8%. Percentage of hypercholesterolemia in high adherence across CC, GC, and GG genotypes were respiratory 40.9%, 37.5%, and 33.3%. **G**. Percentage of hypertriglyceridemia in low adherence across CC, GC, and GG genotypes were respiratory 15.8%, 31.6%, and 15.4%. Percentage of hypertriglyceridemia in high adherence across CC, GC, and GG genotypes were respiratory 95.5%, 100%, and 95.8%

By use of the binary logistic regression model analysis, the interaction between CRY1 polymorphism (rs2287161) and quartiles of AHEI on CVD risk factors was examined. In the models, we included a risk allele genotype (CC genotype), quartiles of AHEI, and interaction between CRY1 polymorphism (rs2287161) and quartiles of AHEI. In the crude model, there was a significant interaction between rs2287161 genotypes and adherence to AHEI on odds of hyper LDL (OR = 1.49, 95% CI =, P for interaction = 0.01) and hypertension (OR = 1.47, 95% CI =, P for interaction = 0.03). After adjusting for age, energy intake, physical activity, FFM, and socioeconomic status, a significant interaction was found between rs2287161 genotypes and adherence to AHEI hyper LDL (OR = 1.94, 95% CI = 1.24–3.05, P for interaction = 0.004), hypertension (OR = 1.80, 95% CI = 1.11–2.93, P interaction = 0.01) and hyperglycemia (OR = 1.56, 95% CI = 0.98–2.47, P interaction = 0.05). There was no significant interaction between rs2287161 genotypes and quartiles of AHEI on other CVD risk factors. Also, we found that adherence to AHEI can decrease the risk of hypo HDL, central obesity, and hypercholesterolemia across CC genotype but their interactions were not significant (Fig. 1).

Additionally, 31.7% of participants in the first quartile, 19.5% of participants in the second quartile, 19.5% of participants in third the quartile, and 29.3% of participants in the last quartile had CC genotype.

## Discussion

This cross-sectional study examined the interaction between the AHEI and CRY1 gene polymorphism on CVDs risk factors in overweight women and women with obesity. The results showed that a negative and strong association was found between adherence to AHEI and the odds of hyper LDL, hypertension, and hyperglycemia across CC genotype. Generally, adherence to AHEI modified the association of the rs2287161 genotypes with the odds of CVDs risk factors such as hyper LDL, hyperglycemia, hypertension, hypo HDL, central obesity, and hypercholesterolemia in the CC genotype.

In the study, we found that the CC genotype had mean higher FBS, ALT, BMI, and FMI and lower HDL and pulse with statistically significant differences. Several studies have presented the crucial role of the CRY gene via the inflection on several chronic diseases. A large sample study that is part of a national health survey in the adult-aged ≥ 30 years showed that the CRY1 genetic variants have a role in elevated blood pressure and high triglyceride [22]. Furthermore, a clinical trial research on mice found that lacking the core clock components CRY1 increases salt sensitive hypertension [41]. A cohort study in the Mediterranean and North American showed that HOMA-IR and fasting insulin had a significant association with CRY1 in risk allele genotype [24]. Tow case control study on adults indicated that CRY1 was associated with obesity and abdominal obesity [20, 42]. Moreover, a study found CRY-deficient mice had decreased glucose tolerance and induced hyperglycemia [43].

A finding of this study indicated that adherence to AHEI in CC genotype decreased the hyper LDL, hypertension, and hyperglycemia with statistically significant differences and reduce hypo HDL, central obesity, and hypercholesterolemia without statistically significant differences. A case cohort study found that tendency to the negative association between adherence to AHEI and diabetes in European populations [44]. Previous studies reported the AHEI was associated with lipid profiles and hypertension [16, 45].

However, the interaction between diet and genes is rarely considered, thus, we investigated the interaction between the AHEI and CRY1polymorphism on cardiovascular risk factors. Although several studies have assessed the interaction of CRY and diet, such as obesity, diabetes, and insulin resistance, no research has been shown to evaluate the interaction between AHEI and CRY1 polymorphism. A cohort study in the Mediterranean and North American indicated that the CRY1 polymorphism had a significant interaction with carbohydrate intake for HOMA-IR and fasting insulin [24]. A study showed that when cryptochrome deficient mice were tested with a high-salt diet, they had hypertension due to irregular synthesis of the mineralocorticoid aldosterone by the adrenal gland [41]. A study on mice found that CRY1 deficiency had an interaction with the high-fat diet and can lead to increased obesity as a result of increased insulin and accumulation of fat in white adipose tissue [46]. Also, a research showed that high fat diet can influence mice with CRY1 deficiency and induct resistance to obesity [31].

It seems that the mechanism of this interaction between the CC genotype and adherence to AHEI with a reduced risk of CVDs risk factor might be due to the contribution of CRY1 in the regulation of steroidogenesis and gluconeogenesis [47]. CRY1 deficiency led to glucose intolerance and high levels of circulating corticosterone which indicates a decrease in the suppression of the hypothalamic-pituitary-adrenal (HPA) axis along with improved glucocorticoid transactivation in the liver [43, 47].On the other hand, an AHEI includes components such as fruits, vegetables, and unsaturated fatty acids that can have positive effects on fat and glucose metabolism [48–51]. According to the studies mentioned above and considering the epigenetic mechanisms, it seems that following a healthy diet can affect the expression of genes involved in the circadian rhythm such as CRY1 [27].

The strength of this study is that it is the first research to estimate the interaction between CRY1 polymorphism and AHEI on the odds of CVDs risk factors in participants, and it was a community-based study. However, our study has some limitations. The cross-sectional design of the study, cannot determine the mechanism of the association between AHEI and the rs2287161 genotype. Also despite controlling several confounding variables in our study, the possible influence of remaining confounders should be considered.

## Conclusion

The results of this study indicate that adherence to AHEI can reduce the odds of hyper LDL, hypertension, and hyperglycemia in the CC genotype. However, the mechanism of interaction between AHEI and CC genotypes is not clearly understood. The results of our study suggest that dietary intake, gene types, and their interactions must be considered in CVDs risk factors evaluation.

## Data Availability

The data that support the findings of this study are available from Khadijeh Mirzaei but restrictions apply to the availability of these data, which were used under license for the current study, and so are not publicly available. Data are however available from the authors upon reasonable request and with permission of Khadijeh Mirzaei.

## Abbreviations

CRY1: Cryptochrome circadian clocks 1
ANOVA: Analysis of variance
ANCOVA: Analysis of covariance
BIA: Bioelectrical Impedance Analyzer
BMI: Body mass index
CVDs: Cardiovascular diseases
DNA: Deoxyribonucleic acid
FFQ: Food frequency questionnaire
GLM: General linear model
GOD/PAP: Glucose oxidase phenol 4-aminoantipyrine peroxidase
GPOPAP: Glycerol-3-phosphate oxidase phenol 4-aminoantipyrine peroxidase
H-ORAC: Hydrophilic oxygen radical absorbance capacity
HDL: High-density lipoprotein
LDL: Low-density lipoprotein
hs-CRP: Hypersensitive C-reactive protein
FBS: fasting blood sugar
TG: Triglycerides
T2D: Type 2 diabetes
WC: Waist circumference
AST: Aspartate aminotransferase
ALT: Alanine aminotransferase
SBP: Systolic blood pressure
DBP: Diastolic blood pressure
BFM: Body fat mass
FFM: Fat free mass
SMM: Skeletal muscle mass
WHR: Waist height ratio
FMI: Fat mass index
FFMI: Fat free mass index
PCR–RFLP: Polymerase chain reactions–restriction fragment length polymorphism
